# ^18^F-FDG PET radiomics model for predicting TARE response in patients with colorectal cancer liver metastases

**DOI:** 10.1007/s11604-026-01949-z

**Published:** 2026-01-28

**Authors:** Osman Melih Topcuoglu, Onur Tuncer, Muge Oral, Ayşegul Gormez, Turkay Toklu, Nalan Alan Selcuk

**Affiliations:** 1 Department of Radiology, Yeditepe University Hospitals, 34718 Kosuyolu, Istanbul, Turkey; 2 Department of Radiology, Medical School, University of Wisconsin-Madison, 600 Highland Avenue Madison, Wisconsin, WI 53792 USA; 3 Department of Nuclear Medicine, Yeditepe University Hospitals, 34718 Kosuyolu, Istanbul, Turkey

**Keywords:** Colorectal carcinoma, Liver metastasis, Machine learning, Radioembolization, TARE

## Abstract

**Purpose:**

Predicting treatment response in patients with colorectal cancer liver metastases (CRCLM) who have undergone transarterial radioembolization (TARE) based on pre-procedural fluorine-18-fluoro-deoxy glucose positron emission tomography (^18^F-FDG PET) radiomics and clinical information.

**Materials and methods:**

Patients with CRCLM who underwent TARE, between March 2015 and May 2025, were consecutively included. Largest tumors were segmented semiautomatically using pre-procedural ^18^F-FDG PET images. Radiomics features were extracted, clinical information were collected. Two datasets were created comprising radiomics-only and clinico-radiomic features. Datasets were divided 60:40 for training and testing. Top 5 features were selected based on feature importances. Random Forest, Extreme Gradient Boosting, Logistic Regression models were trained. Test-set area under the curves (AUCs) for predicting post-treatment target lesion local progression were calculated and compared using DeLong’s test. Sensitivity, specificity, accuracy and F1 scores were calculated at the optimal cut-offs.

**Results:**

Seventy-four patients out of 96 patients were included. Top five selected features in the radiomics-only dataset were Coarseness, IMC1, Zone Entropy, Size-Zone Non-Uniformity, and Strength. In the clinico-radiomic dataset, AST and ALT levels were substituted among the top five features. Radiomics-only features demonstrated AUCs ranging from 0.90 (95% CI 0.71–1) to 0.81 (95% CI 0.51–1) in the test-set while clinico-radiomics dataset AUCs varied between 0.88 (95% CI 0.51–1) and 0.84 (0.62–1).

**Conclusion:**

^18^F-FDG PET radiomics based models can predict the local response to TARE in patients with CRCLM, in this series.

## Introduction

Radiomics is based on radiological imaging in contrast to the other “omics” as genomics and proteomics which depend on invasive biopsy or molecular assays. It enables the extraction of pertinent features from medical images to effectively capture the heterogeneous characteristics of regions of interest [1]. By capturing details invisible to the naked human eye, it has the potential to unveil associated biological, phenotypic, and genotypic features of tumors, thereby enabling the possibility of making clinical predictions. In the literature radiomics-based methodologies are widely employed across diverse imaging modalities to predict treatment response for various cancers [2–4]. When considering particularly for the response prediction in transarterial radioembolization (TARE), studies centered on predicting treatment responses after TARE, primarily focused on hepatocellular carcinoma (HCC) [5]. In the context of colorectal cancer liver metastases, radiomics-based response evaluation is primarily directed toward systemic therapy or surgical interventions [6]. In the current study, the aim was to build a machine learning (ML) model using pre-procedural fluorine-18-fluoro-deoxy glucose positron emission tomography (^18^F-FDG PET) based radiomics and clinical information to predict local lesion response after TARE in patients with colorectal cancer liver metastases (CRCLM).

## Material and methods

### Patient population

**Fig. 1:**
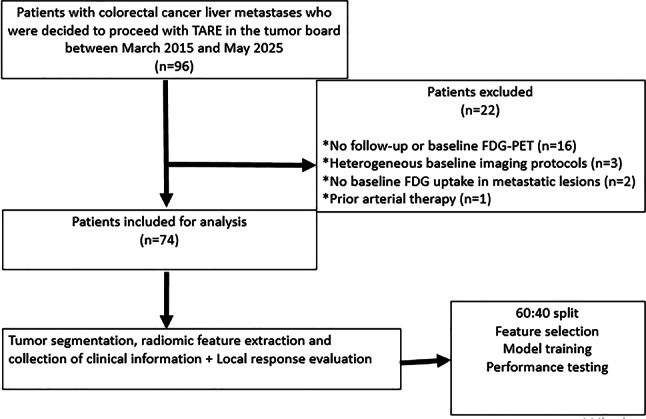
Patient selection flowchart

**Fig. 2:**
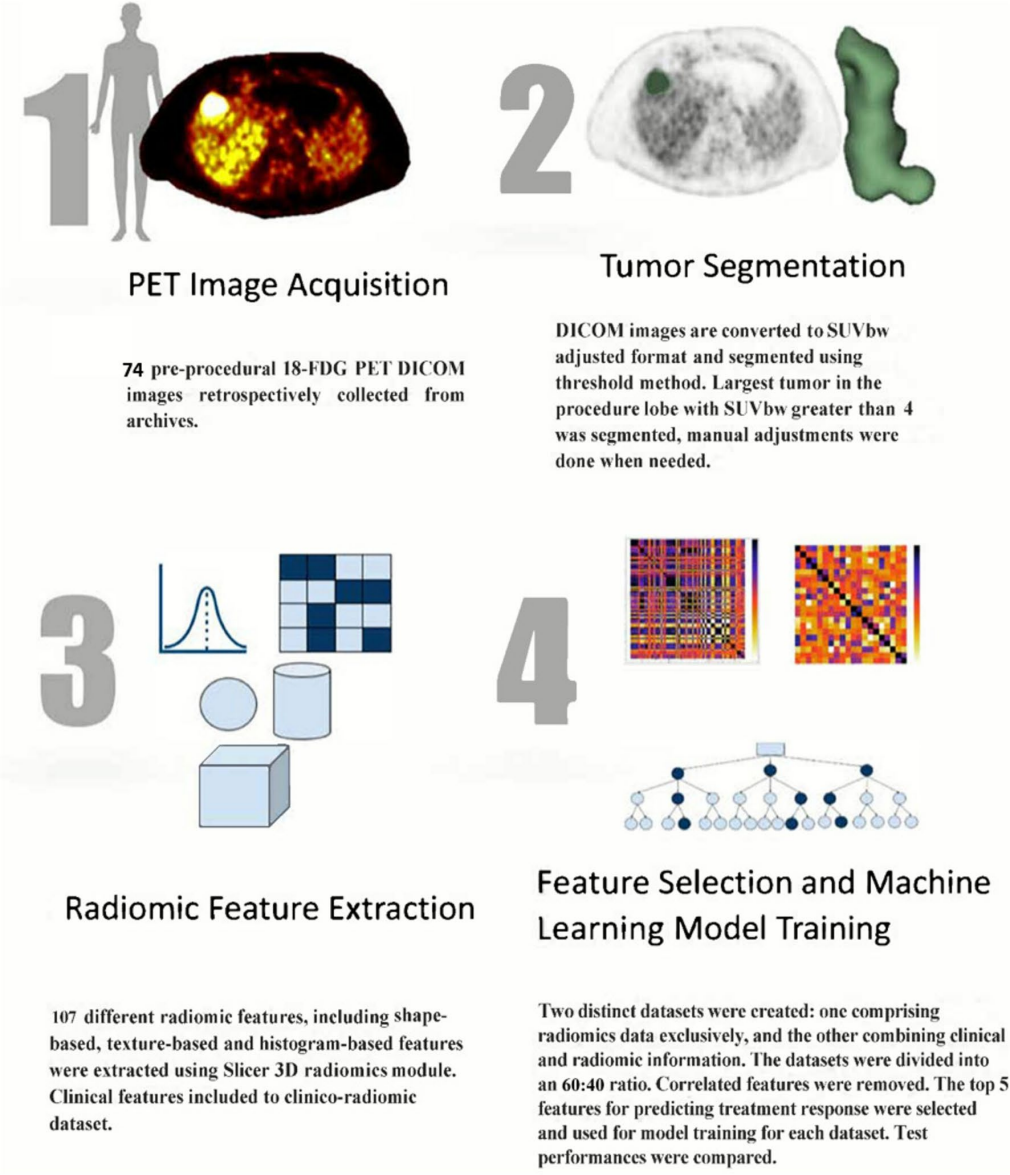
Study plan

Institutional Review Board approved this single centre retrospective study. Written informed consent was waived for this type of study. Radiological, histopathological, and clinical information including age, sex, yttrium-90 dose, primary tumor location, treated liver lobe, model for end-stage liver disease (MELD) score, laboratory values were recorded from the hospital’s electronic archive. Between March 2015 and May 2025, patients with unresectable CRCLM who underwent TARE in a single institution, were consecutively included. All treatments were decided at the multidisciplinary tumor board. Patients without baseline ^18^F-FDG PET scan prior to TARE procedure, patients without follow-up imaging, patients with CRCLM which did not show ^18^F-FDG affinity, patients with prior intraarterial therapy as chemoembolization or radioembolization were excluded (Figs. 1 and 2).

### ^18^F-FDG PET imaging

All ^18^F-FDG PET images were obtained from the same PET CT scanner (Discovery 710, GE Healthcare, Milwaukee, WI, USA) with integrated 64-slice CT, high resolution, time-of-flight (TOF) function, and LYSO crystal. After intravenous injection of a radiopharmaceutical with an average activity of 300–400 MBq, patients were fixed supine on the bed of the PET scanner 45–50 min after injection. CT and PET images were obtained from the vertex region to mid-thigh. Pre-procedural PET CT scans were performed up to 3 months prior to TARE and post-procedural PET CT images were obtained at 3 months following TARE.

### TARE procedure

All procedures were performed via standard right trans-femoral approach. Technetium-99 m macro-albumin aggregate (99mTc-MAA) infusion was made from the placed microcatheter either from the right or the left hepatic artery. Proceeding to TARE was decided according to the single photon emission computed tomography (SPECT–CT) findings. Yttrium 90-labeled microspheres (Therasphere™; Boston Scientific, Marlborough, Massachusetts) were injected from the identical microcatheter localization. Lobar or sequential bilobar treatment was made. Antibiotic prophylaxis was not used. All procedures were carried out by an interventional radiologist with more than 10 years of individual experience in intra-arterial procedures. Dosimetry was planned using a multi-compartment model. Delineation of tumors and image assessments were performed on Simplicit90Y® software (Mirada Medical Ltd. Oxford, UK). Personalized dosimetry was used in all patients as per standard of care.

### Response evaluation and definition of outcomes

Post-procedural ^18^F-FDG PET CT images were evaluated by both a nuclear medicine physician with more than 20 years of experience and an interventional radiologist with more than 15 years of experience in oncological imaging in a blinded manner. Primary outcome was target lesion local progression at 3-month follow-up ^18^F-FDG PET CT. Each target lesion was assessed seperately according to positron emission tomography response criteria in solid tumors (PERCIST) to determine treatment response. Assessments were classified as complete response-CR (complete resolution of ^18^F-FDG uptake within the target lesions), partial response-PR (reduction of a minimum of 30% in target tumors), progressive disease-PD (> 30% increase in ^18^F-FDG uptake in target tumors or new FDG avid lesions) and stable disease-SD (no CR, no PR or no PD). Outcomes were binarized as non-viable tumor and viable tumor. Non-viable tumor tissue and viable tumor tissue were defined as CR and PR + SD + PD, respectively.

### Segmentation and radiomics feature extraction

**Fig. 3:**
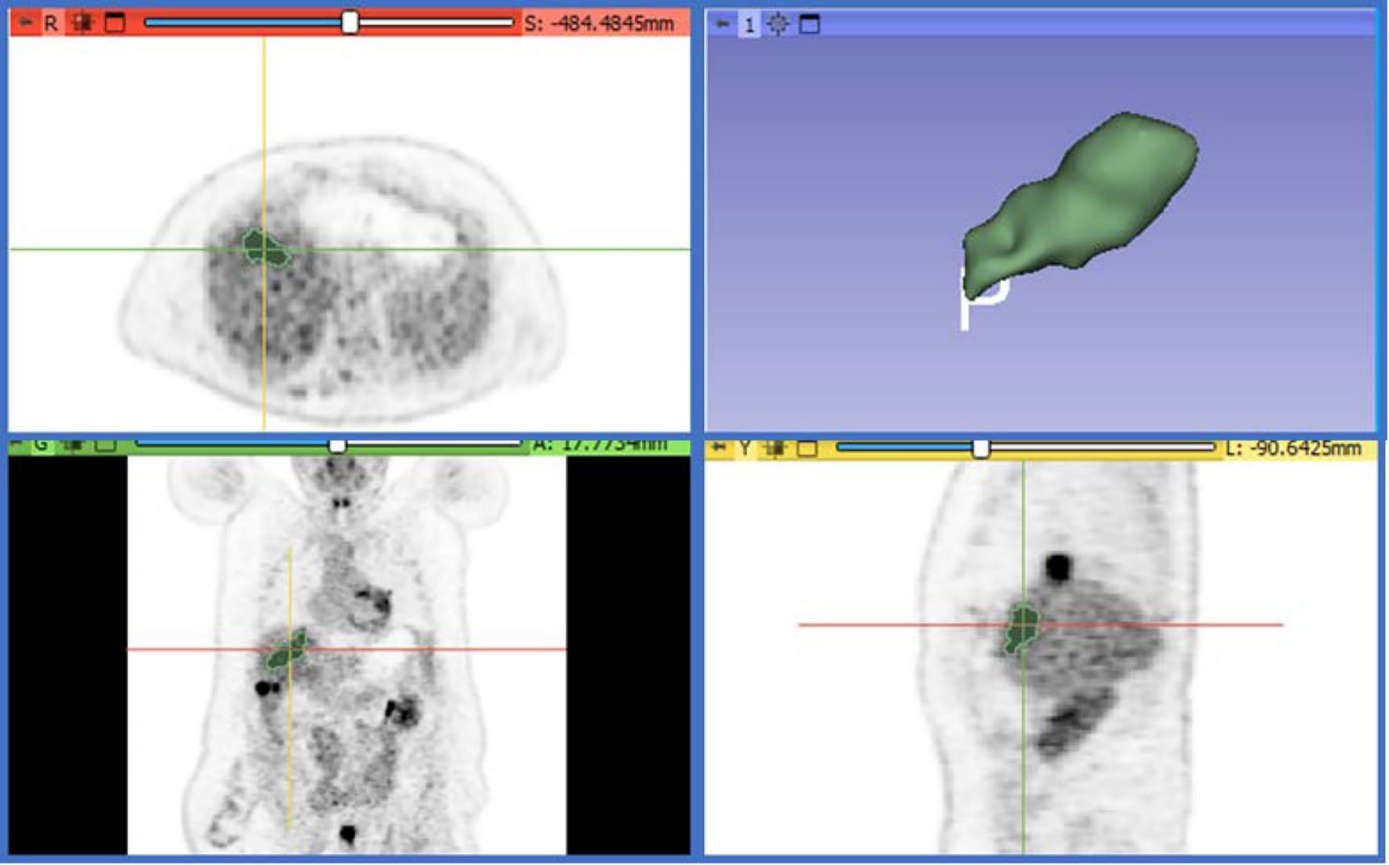
Tumor segmentations. All tumor segmentations were performed using 3D Slicer software. All ^18^F-FDG PET images were converted to SUVbw adjusted values. The largest lesions in the procedure lobe were first segmented automatically using the threshold method. Tumors with SUVbw greater than 4 were automatically selected within the largest tumor. In instances when there are adjacent tumors and automatic segmentation yielded unsatisfactory results, such as overlapping borders with adjacent tumors, manual corrections were made through three different planes. Finally, the segmented volume underwent radiomic feature extraction. *SUVbw* Standardized Uptake Values body-weight adjusted

Accordingly to previously published PET radiomics guidelines [7], all segmentations were conducted within 3D Slicer, an open source software tool designed for medical image segmentation (v.4.11.2, https://www.slicer.org) [8]. Initially, preoperative ^18^F-FDG PET CT images were converted to body weight adjusted standardized uptake values (SUVbw). The segmentation process primarily employed the threshold method, selecting regions of interest with greater than 4 SUVbw [9, 10] blinded to any patient information. The largest lesion within the treated liver lobe was targeted (Fig. 3). Following the completion of the segmentation process, radiomic features were extracted through the utilization of the 3D Slicer Radiomics module, which is image biomarker standardization initiative (IBSI) compliant PyRadiomics based software platform [11]. Voxel dimensions were uniformly resampled to 1 × 1 × 1 voxel size, the binwidth were adjusted to 0.4 SUVbw and symmetrical gray-level co-occurrence matrix (GLCM) were enforced. For every segmented volume, we extracted a total of 107 features including 18 first order, 14 shape, 24 GLCM, 16 Gy level size zone matrix (GLSZM), 16  Gy level run length matrix (GLRLM), 14 Gy level dependence matrix (GLDM), and 5 neighboring gray tone difference matrix (NGTDM) features.

### Feature selection and machine learning model training

Radiomic-only and clinico-radiomic datasets were created using 107 radiomic features (supplementary material) and additional 16 clinical features (demographics, lab values, dosimetry, tumor location) (Table 1). The Scikit-Learn library in Python 3.12 was employed for feature selection and classification. Each dataset were divided into 60% for training and 40% for testing sets. Numeric values were normalized using the StandardScaler. Subsequently, an analysis of pearson correlation was performed using the training set, eliminating feature pairs with more than 80% correlation. Following this, the SelectKBest method, utilizing linear regression as the scoring function, was employed for feature selection through fivefold cross-validation. The top 5 radiomic features were identified according to mean feature importances. These features were then used to train 3 commonly used classifiers: Random Forest (RF), Extreme Gradient Boosting (XGB), Logistic Regression (LR). Performence metrics for each model were evaluated on the test-set.

**Table 1:** Study Cohort characteristics

	TARE response		
	Non-viable tumor tissue	Viable tumor tissue	*P* value
Patient number	24	50	
Age (years)	66.5 (66–69.5)	67 (56–73)	0.881
90Y dose (Gy)	191.5 (150–208)	169 (134.5–200)	0.506
Sex			0.432
M	14	37	
F	10	13	
Liver Lobe			0.791
Right	14	38	
Left	8	9	
Bilobar	2	3	
MELD score	7 (6–7)	7 (6.5–10.5)	0.174
ALT	25.5 (24–26)	27.5 (22–37)	0.079
AST	45 (31–45)	45 (34–82.50)	0.123
Sodium	139.5 (139.5–139.5)	139.5 (138–140)	0.722
Creatinine	0.71 (0.61–0.74)	0.78 (0.63–1.08)	0.206
INR	1.04 (0.93–1.10)	1.04 (0.99–1.19)	0.414
PT	13.4 (12.7–13.9)	13.4 (12.85–14.75)	0.499
Total Bilirubin	0.48 (0.47–0.48)	0.48 (0.47–0.73)	0.279
Albumin	3.85 (3.85–4.30)	3.85 (3.40–4.08)	0.341
BUN	13.5 (12–13.50)	13.5 (10–19.50)	0.594
Primary tumor localization			0.351
Right colon	7	8	
Left colon	17	42	

*TARE* transarterial radioembolization, *MELD* model for end-stage liver disease, *Lab* laboratory

### Statistical analysis

Mann–Whitney U test was performed for numerical, Fisher’s exact test was used for categorical features using SPSS v.23 (IBM, Armonk, New York). Missing laboratory values were inputed with median values. Statistical significance was set at two-tailed *p* < 0.05. ROC curves in test sets were drawn. The optimal cutoff points were determined based on the Youden index, and subsequently sensitivity, specificity, accuracy and F1 score were calculated for each machine learning classifier and dataset. DeLong`s test using R version 4.2.0 (2022-04-22) was utilized for confidence interval calculation and comparing area under curves (AUCs) of datasets.

## Results

In this study we applied STARD guidelines (supplementary material). Of the 96 patients, a total of seventy-four patients met the inclusion criteria (Fig. 1). The primary colorectal cancer was operated in 57 patients (77%). Distant extrahepatic metastases were present in 41 patients (55.4%). These metastases affected lymph nodes, bones and the pulmonary parenchyma. The study cohort was summarized in Table 1. Response to TARE at 3-month follow-up was CR (n = 24), PR (n = 31), PD (n = 17) and SD (n = 2). After dichotomizing the response groups into non-viable tumor tissue and viable tumor tissue, the median ages were 66.5 years (IQR, 66–69.5 years) and 67 years (IQR, 56–73 years), respectively. The median delivered tumor doses were 191.5 Gy (IQR, 150–208 Gy) in the first group and 169 Gy (IQR, 134.50–200 Gy) in the second group. Both groups exhibited a predominance of left colon primary tumors and right lobar procedures. No statistically significant differences were observed in demographic, clinical and laboratory variables between response groups as well as training and test groups (Tables 1 and 2). Our radiomics quality score 1 and 2, a scoring system for showing validity of methodology, analysis, and reporting of radiomics studies, were 11 and 35 respectively (supplementary material). In the radiomics-only dataset, the top 5 radiomic features were ranked as: Coarseness, Informational Measure of Correlation 1 (IMC1), Zone Entropy (ZE), Size-Zone Non-Uniformity (SZN), and Strength. In the clinico-radiomic dataset, the top 5 features were ranked as follows: AST, Coarseness, IMC1, ZE, and ALT.

**Table 2:** Training-test Cohort characteristics

	Training-test cohorts		
	Training cohort (%60)	Test cohort (%40)	*P* value
Number of patients	44	30	
Age (years)	67 (56–72)	67 (62–70)	0.964
90Y dose (Gy)	167 (148–200)	193 (127–208)	0.445
Sex			0.799
M	32	21	
F	12	9	
Liver Lobe			0.948
Right	31	22	
Left	11	7	
Bilobar	2	1	
MELD score	7 (6–8)	7 (6–11)	1
Lab. values			
ALT	26 (17–38)	26 (26–33)	0.342
AST	45 (29–82)	45 (35–45)	0.797
Sodium	139.5 (139–141)	139.5 (138–139.5)	0.116
Creatinine	74 (58–92)	74 (67–110)	0.753
INR	101 (96–114(	101 (99–116)	0.763
PT	134 (128–146)	134 (124–144)	0.377
Total Bilirubin	48 (47–74)	48 (47–55)	0.437
Albumin	43.5 (37–44)	43.5 (43.5–279)	0.154
BUN	13.5 (11–17)	13.5 (10–29)	0.401
Primary tumor localization			0.09
Right colon	7	8	
Left colon	37	22	
Treatment response			0.891
Viable tumor (CR)	14	10	
Non-viable tumor (PR + PD + SD)	30 (19 + 10 + 1)	20 (12 + 7 + 1)	

*INR* international normalized ratio, *PT* Prothrombin time, *BUN* Blood Urea Nitrogen, *TARE* transarterial radioembolization, *MELD* model for end-stage liver disease, *Lab* laboratory

**Fig. 4:**
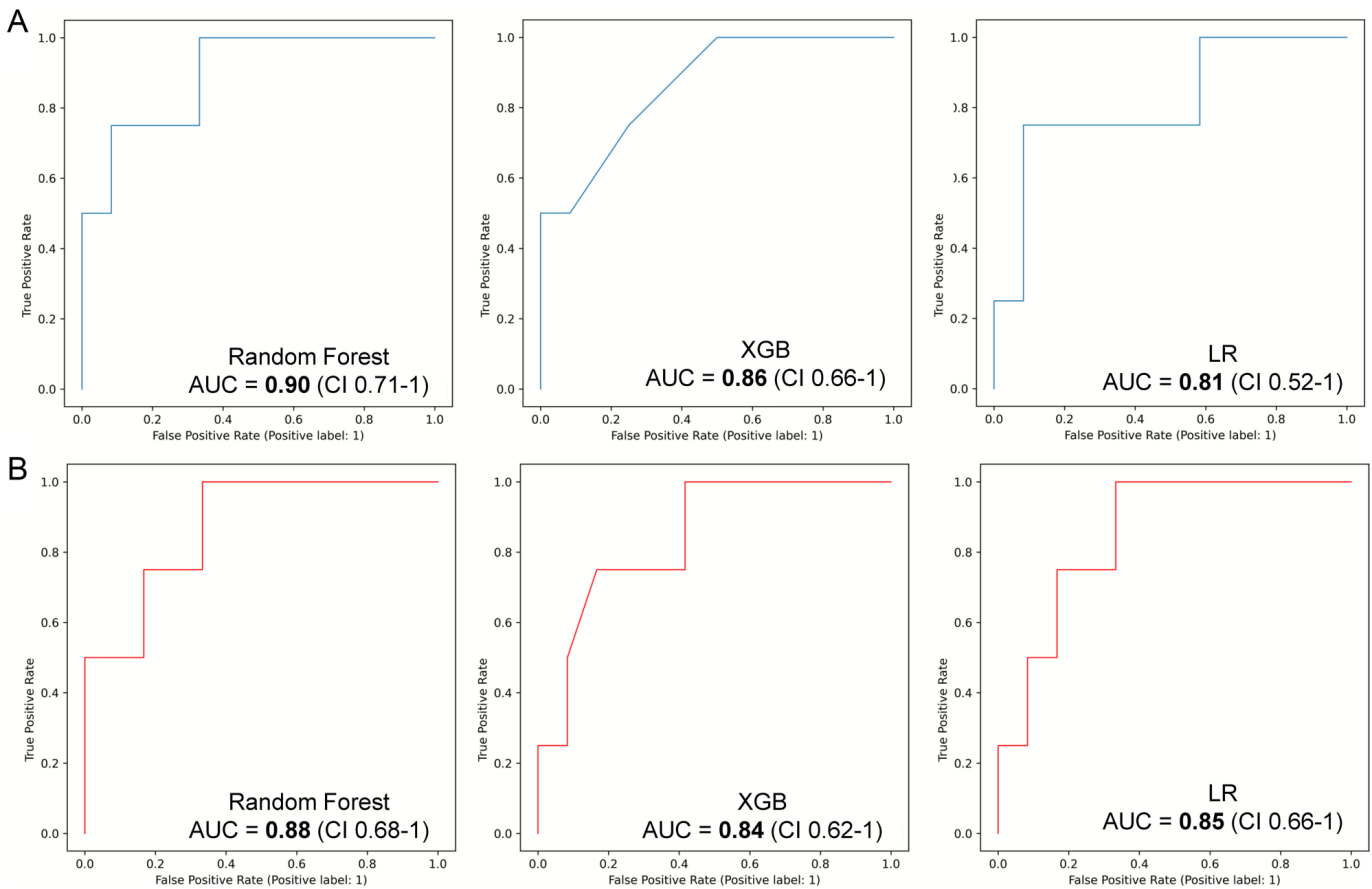
Test set, ROC curves of 3 different machine learning models using the radiomics-only features (**A**) and the clinico-radiomic features (**B**). *ROC* Receiver Operating Characteristics, *AUC* Area Under the Curve, *XGB* Extreme Gradient Boosting

In the radiomics-only dataset, the AUCs of RF, XGB and LR were 0.90 (95% CI 0.71-1), 0.86 (95% CI 0.66-1), 0.81 (95% CI 0.52-1) respectively (Fig. 4A). In the clinico-radiomic dataset, the AUCs of RF, XGB and LR were 0.88 (95% CI 0.68-1), 0.84 (95% CI 0.62-1), 0.85 (95% CI 0.60-1) respectively (Fig. 4B). Delong’s test did not show significant AUC difference between 2 datasets for any of the classifiers. The performance metrics at the optimal threshold for both datasets are presented in Table 3.

**Table 3:** Performance metrics of each model with 2 different datasets

	Random forest			XGB			Logistic regression		
	Radiomics only	Clinicoradiomic	P*	Radiomics only	Clinicoradiomic	P*	Radiomics only	Clinicoradiomic	P*
AUC (%95 CI)*	0.90 (0.71–1)	0.88 (0.68–1)	0.76	0.86 (0.66–1)	0.84 (0.62–1)	0.85	0.81 (0.52–1)	0.85 (0.66–1)	0.64
Sensitivity (%)	100	100		50	75		75	100	
Specificity (%)	67	67		100	83		92	67	
Accuracy (%)	75	75		88	81		88	75	
F1 score	0.67	0.67		0.67	0.67		0.75	0.67	

*AUC* Area Under the Curve, *CI* Confidence Interval, *XGB* Extreme Gradient Boosting

* The 95% confidence intervals (CIs) for the area under the curve (AUC) of each model were calculated, followed by a comparison between the AUCs of Radiomics and Clinicoradiomics models using the DeLong`s method

## Discussion

In this study, TARE local response prediction capabilities of ML models based on pre-procedure ^18^F-FDG PET radiomics and clinical features were assessed in patients with CRCLM. The response groups were categorized into non-viable and viable tumor tissue. Three distinct classifiers (Random Forest, Extreme Gradient Boosting, Logistic Regression) with radiomics-only features demonstrated AUCs ranging from 0.90 (95% CI 0.71-1) to 0.81 (95% CI 0.51-1) in an un-seen test set. Using the clinico-radiomics dataset classifier AUCs varied between 0.88 (95% CI 0.51-1) and 0.84 (0.62-1). In both datasets, RF classifier achieved 100% sensitivity at the optimal threshold. This study represents a promising preliminary analysis of our methodology. A larger, multicenter study confirming the utility of our methodology could significantly impact patient selection in the clinical setting. In other words, before deciding to include patients in the TARE schedule, oncology tumor boards could anticipate the benefit provided by TARE.

Previous studies investigated TARE response prediction predominantly using MR or CT based radiomics in HCC patients [5, 12–19]. Ince et al. [5] reported significant performance difference regarding predicting TARE response in patients with HCC when MR-based radiomic features included to the clinical features. However, Marinelli et al. [12] reported that MR-based radiomic features yielded higher AUC values for response prediction compared to clinical data, in HCC patients undergoing treatment with resin spheres, however, the difference was not significant. The performances of ML models were not significantly altered when the clinical features were combined with the radiomics data, in the present study.

PET-based radiomics were also utilized in the literature for the purpose of TARE response anticipation [20–23]. When comparing our findings with those of previous PET-based radiomics studies that evaluated TARE response [20–23], it is important to emphasize several key differences. Unlike most earlier investigations, which focused predominantly on HCC and often incorporated whole-liver radiomics or post-therapy 90Y PET metrics, our study uniquely evaluates pre-procedural 18F-FDG PET radiomics in patients with CRCLM. Previous studies have reported varying predictive performances, with AUC values generally ranging from moderate to high. However, these studies frequently used heterogeneous imaging protocols, multiple scanners or radiomics derived from post-treatment imaging, which may influence feature stability and model performance. In contrast, our use of a single scanner and standardized acquisition protocol probably reduced technical variability, which may explain the high test-set AUCs observed. Furthermore, the distinct tumour biology of CRCLM compared with HCC likely contributes to differences in the identified radiomic predictors. These methodological and biological differences may explain the discrepancy between previous findings and the present results, and highlight the need for multicenter, disease-specific validation. Blanc-Durand et al. [22] reported that pre-treatment whole-liver ^18^F-FDG PET could predict overall survival and hepatic progression free survival in patients with HCC. Wei et al. [23] published that their model utilizing radiomics features extracted from post-therapy ^90^Y PET and mean absorbed dose, reached an AUC of 0.803 (95% CI 0.702–0.758) for predicting lesion response in HCC patients. They emphasized that despite the noisy nature of post TARE PET images, combined ML models could predict disease progression.

Radiomics is an emerging and rapidly growing field of research area in radiology [24]. There has been a notable increase in the number of studies reporting outcome predictions through the utilization of radiomics. However, it is imperative to acknowledge certain limitations observed in these studies. The majority of reported studies exhibit variations in machine learning pipelines, the presence of an unseen test set, and imaging protocols. Moreover, the utilization of multiple scanners for image acquisition, heterogenous patient population, and small sample sizes are also commonly seen. The reported high scoring performance metrics might be over optimistic, considering their lack of generalizability and reliability. Nevertheless, radiomics could still provide helpful information in clinical practice. A recent meta-analysis investigating early prediction of radioembolization treatment response with radiomics, have shown a pooled sensitivity and specificity of 83.5% (95% CI 76%-88.9%) and 86.7% (95% CI 78%-92%), respectively [25]. In the current study, when clinical features were included, three of the five radiomic features remained at the top, and radiomic features alone showed similar performance to the combination of radiomic and clinical features in predicting response to TARE, underscoring the reliability of radiomic features. The top five radiomic features identified in the present models may reflect the underlying biological characteristics of the tumor. ‘Coarseness’ quantifies the rate of intensity change across neighboring voxels and is often associated with the degree of intratumoral heterogeneity — coarser textures may correspond to more heterogeneous metabolic activity or necrotic components. IMC1 reflects the complexity and non-uniform relationships among grey-level intensities. Lower IMC1 values have been associated with disorganised microstructural patterns and aggressive tumor behaviour. ZE measures the randomness of homogeneous zones within the tumor and increases with metabolic heterogeneity, which may be associated with uneven cellularity or variable perfusion. SZN describes variability in zone sizes — higher SZN values indicate irregular clusters of similar intensity and may reflect heterogeneous architecture, such as the presence of both viable and necrotic regions. ‘Strength’ captures the perceptual prominence of structured patterns within the lesion. Higher values may be associated with well-defined, spatially coherent metabolic patterns, whereas lower values reflect disorganised tissue structure. While these relationships are not direct histological equivalents, they imply that predictive radiomic features could act as surrogates for tumor heterogeneity, cellular disorganisation, and microstructural complexity.

In a recent CT based study by Roll et al. [26] including patients with CRCLM, found AUC of 0.75 (95% CI 0.48-1). The authors made the radiomics analysis from enhanced CT images obtained from different scanners under different CT parameters. Our study is unique in being ^18^F-FDG PET radiomics-based TARE response prediction focused solely on patients with CRCLM. Also we utilized a uniform image acqusition, evaluated radiomics data obtained from the same PET scanner with the same imaging protocol for all patients leading to less data heterogenity.

This study has several limitations. First of all, external validation was not performed, and it is unclear whether the results of this study possess generalizability applicable to clinical practice. A relatively small sample size and retrospective nature of the study were the other major drawbacks. The single-center, retrospective design of the study inherently restricts the generalizability of the findings. As all PET/CT examinations were performed on the same scanner using a uniform acquisition and reconstruction protocol, the identified radiomic features may reflect scanner- or protocol-specific characteristics rather than universally reproducible biomarkers. PET/CT radiomics is sensitive to variations in technical parameters and differences in scanners, reconstruction algorithms or segmentation workflows across institutions could affect feature stability and model performance. Therefore, external validation in larger, multicenter cohorts with heterogeneous imaging platforms and standardized acquisition protocols is essential to confirm the robustness and reproducibility of our proposed models. Furthermore, our analysis did not include multiple segmentations with different segmentators, which means that the reproducibility of features was not thoroughly assessed. Lastly, the 3-month follow-up evaluation may have led to misinterpretation such as pseudoprogression due to the early timing of the assessment.

In conclusion, the current study demonstrated the potential of baseline 18F-FDG PET radiomics for predicting TARE response in patients with CRCLM. These findings could inform future studies using larger, standardised datasets, which may lead to the development of a clinical guidance tool for patient selection and personalised medicine.
